# Why Do Age and Bias Matter? Historical, Social, and Biological Impacts Across Generations of LGBTQ Older Adults

**DOI:** 10.1093/geroni/igaa057.2612

**Published:** 2020-12-16

**Authors:** Charles Emlet, Karen Fredriksen Goldsen

## Abstract

We do not live in a stagnant world, politically, historically or socially - neither are the life experiences of lesbian, gay, bisexual, and transgender (LGBTQ) older adults. Their physical, psychological and social lives are impacted by their present and past historical contexts and generational identity, as well as the sociopolitical environment, social pressures, support and experiences of biases. This symposium draws upon data from Aging with Pride: National Health, Aging and Sexuality/Gender Study, the first longitudinal study of LGBTQ older adults in the United States, in order to examine how environmental, interpersonal and intrapersonal structures impact the lives of the three oldest generations of LGBTQ adults. Emlet and colleagues examine the generational and historical forces across the life course among 205 transgender older adults in order to illuminate how historical context and gender influences social life and physical health. Prasad and colleagues explore differences between various constructs of bias including lifetime discrimination, victimization, everyday discrimination and microaggressions on LGBTQ older adults and how those experiences impact depression, general health and quality of life. Lastly, Hoy-Ellis and colleagues explore how life course predictors including interpersonal violence, legal marriage, and identity management are associated with allostatic load (AL) in a sample of 317 LGBTQ older adults who range in age from 50-97. We will address the intersectionality of generational identity, experiences of bias and biomarkers, as well as the implications for policy and practice.

